# Empirical evidence about recovery and mental health

**DOI:** 10.1186/s12888-015-0678-4

**Published:** 2015-11-14

**Authors:** Mike Slade, Eleanor Longden

**Affiliations:** King’s College London, Health Service and Population Research Department (Box P029), Institute of Psychiatry, Psychology & Neuroscience, Denmark Hill, London, SE5 8AF UK; Institute of Psychology, Health and Society, University of Liverpool, Block B, 2nd Floor, Waterhouse Building, Liverpool, L69 3GL UK

**Keywords:** Mental health, Recovery, Science, Evidence, Prognosis, Outcome

## Abstract

**Background:**

Two discourses exist in mental health research and practice. The first focuses on the limitations associated with disability arising from mental disorder. The second focuses on the possibilities for living well with mental health problems.

**Discussion:**

This article was prompted by a review to inform disability policy. We identify seven findings from this review: recovery is best judged by experts or using standardised assessment; few people with mental health problems recover; if a person no longer meets criteria for a mental illness, they are in remission; diagnosis is a robust basis for characterising groups and predicting need; treatment and other supports are important factors for improving outcome; the barriers to receiving effective treatment are availability, financing and client awareness; and the impact of mental illness, in particular schizophrenia, is entirely negative. We selectively review a wider range of evidence which challenge these findings, including the changing understanding of recovery, national mental health policies, systematic review methodology and undertainty, epidemiological evidence about recovery rates, reasoning biased due to assumptions about mental illness being an illness like any other, the contested nature of schizophrenia, the social construction of diagnoses, alternative explanations for psychosis experiences including the role of trauma, diagnostic over-shadowing, stigma, the technological paradigm, the treatment gap, social determinants of mental ill-health, the prevalence of voice-hearing in the general population, and the sometimes positive impact of psychosis experience in relation to perspective and purpose.

**Conclusion:**

We propose an alternative seven messages which are both empirically defensible and more helpful to mental health stakeholders: Recovery is best judged by the person living with the experience; Many people with mental health problems recover; If a person no longer meets criteria for a mental illness, they are not ill; Diagnosis is not a robust foundation; Treatment is one route among many to recovery; Some people choose not to use mental health services; and the impact of mental health problems is mixed.

## Background

How should entitlement to disability-related benefits and other social resources be established? Welfare systems tend to be structured on a categorical basis, e.g. Not disabled versus Disabled. For example, Australia's trial National Disability Insurance Scheme requires ‘permanent or likely to be permanent impairment or disability’ as an eligibility criteria. In 2014 a literature review was undertaken by the Centre for Mental Health at the University of Melbourne [1], to inform the policy.

The review examined “*the current state of evidence relating to the impact of psychosocial disability*” (p. 1) in the context of implementing the National Disability Insurance Scheme Act 2013 in Australia. It included a review of international approaches to understanding the concepts of ‘permanent’ and ‘impairment’ in disability legislation, the evidence relating to outcome for three specific diagnoses (schizophrenia, bipolar disorder and depression), and how people living with ‘psychosocial disability’ can be supported.

Where does the review sit in terms of quality? Evidence synthesis (i.e. a literature review) involves the integrating of available evidence to reach a justified conclusion. The most rigorous review methodology is called a systematic review, in which the aim is to synthesise all, or nearly all, available evidence relating to a particular question. Other review methodologies also exist [2], and we would position the 2014 review as a ‘systematized review’, i.e. one which includes elements of a systematic review process while stopping short of being a full systematic review.

As a minor point, the 2014 review has not to our knowledge been published as an academic paper, and hence has not been peer reviewed. The peer review process might have highlighted some limitations, such as no rationale being given for the chosen date range (1994 to 2014; thus excluding some of the seminal long-term outcome studies [3–6] finding recovery rates for schizophrenia in excess of 50 %), the use of only one electronic database (PsycInfo), the lack of clarity about how the search terms (which are given) were used, and the lack of clearly stated inclusion/exclusion criteria. However, we mention these points only in passing, mindful that the authors have not positioned their review as systematic, nor claimed it has been peer reviewed. Overall, we view the 2014 review as a robust and good-quality review, which is well written and transparent in its methods, competent within the frame of reference used, and does not go beyond the data. It therefore helpfully illustrates the knowledge contribution of one form of science.

What did the review conclude? Some conclusions (pp.73–75, all quotes verbatim) were:

1. *The judgement of the disability and its likely persistence tends to be made using a standard battery of tests…and/or the opinions of appropriate experts*.
2. *The negative effects of mental illness are, for a large proportion of people, ongoing and pervasive*.
3. *Mental illness is most often not ‘permanent’ in the sense that its effects are not consistent over time, though the pattern of impairment and functioning can persist for many years*
4. *The best predictors of the long-term course of a particular form of mental illness are likely to be the diagnosis itself, with people with schizophrenia tending to experience worse outcomes than people with other disorders, and characteristics of the illness occurring in the early stages.*
5. *The outcomes are likely to be mitigated by many other factors such as access to quality treatment and supports.*
6. *Many people with a psychosocial disability, however, also report having unmet support needs. Their needs might be unmet because no suitable services exist or because the services they are using do not fully meet their needs. Alternatively, the needs of people with mental illness also go unmet because they cannot afford to access services to meet them or because they do not know how to find out about existing services and how to access them.*
7. *Research evidence shows that people with severe mental illness are most often affected to some degree in all areas of their daily living, experiencing difficulties in social and occupational functioning, maintaining a home and completing the tasks of daily living…Many people with a psychosocial disability also experience homelessness…People with schizophrenia seem to be the most severely disabled.*

We summarise these seven conclusions as seven messages:

1. Recovery is best judged by experts or using standardised assessments
2. Few people with mental health problems recover
3. If a person no longer meets criteria for a mental illness, they are in remission
4. Diagnosis is a robust basis for characterising groups and predicting need
5. Treatment and other supports are important factors for improving outcome
6. The barriers to receiving effective treatment are availability, financing and client awareness
7. The impact of mental illness, in particular schizophrenia, is entirely negative.

To re-iterate, the review was well written and transparent in its methods – it represents one form of science. However, we believe that its findings are influenced by embedded assumptions, and there are other types of evidence which lead to alternative conclusions. Although the review aim related to psychosocial disability, its conclusions involve concepts which are becoming debated under the topic of ‘recovery’. The aim of this article is to provide a balancing perspective on what a wider appraisal of evidence tells us about recovery in the context of mental ill-health. Our selective appraisal of a broader range of scientific evidence with different assumptions leads to a different seven messages.

## Discussion

### A note on language

In this article we seek to highlight some contested assumptions. Often these assumptions are contained within language, for example when we talk about ‘cause’ , ‘remission’ , ‘illness’ , ‘patient’ etc.

Language can be unhelpful in hiding these assumptions, and this article is specifically seeking to make relevant assumptions visible – hence we try as far as possible to use neutral language. We specifically use ‘person-first’ language, which avoids terms such as ‘schizophrenic’ as descriptions of individuals.

Language can also of course be unhelpful if it is too convoluted, such as ‘person having experiences which a mental health professional would diagnose as a mental illness’. We therefore use recognised and somewhat but not totally neutral terms (e.g. mental health problems) whilst acknowledging that they remain contested (e.g. some frame their experiences in trauma or spiritual terms).

### Seven evidence-based alternative messages

We now make the scientific case for seven different messages. Our knowledge, and therefore the majority of the evidence we cite, relates primarily to schizophrenia. The issues overlap with the other two diagnoses – bipolar disorder and depression – covered in the 2014 review.

### Message 1: Recovery is best judged by the person living with the experience

The meaning of recovery in the context of mental health problems is changing. The old meaning – which we term ‘clinical recovery’ – has emerged from professional-led research and practice. It has four key features:

1. Recovery is an outcome or a state, generally dichotomous – a person is either ‘in recovery’ or ‘not in recovery’
2. It is observable – in clinical language, it is objective, not subjective
3. It is rated by the expert clinician, not the patient
4. The definition of recovery does not vary between individuals

Various definitions of clinical recovery have been proposed by mental health professionals. A widely-used definition is that recovery comprises full symptom remission, full or part-time work or education, independent living without supervision by informal carers, and having friends with whom activities can be shared, all sustained for a period of two years [7]. Although not a term used in the definition, this could be summarised as being ‘normal’.

The definition of clinical recovery does not vary across individuals, which means it can be defined, measured and investigated in empirical studies. The 2014 review illustrates this approach. However, deep assumptions about normality are embedded in clinical recovery:

“*This kind of definition begs several questions that need to be addressed to come up with an understanding of recovery as outcome: How many goals must be achieved to be considered recovered? For that matter, how much life success is considered “normal”?*” [8] (p.5)

A different understanding of recovery has emerged from the mental health service user and survivor movement. This second meaning can be called ‘personal recovery’. In contrast to clinical recovery, personal recovery:

1. Is a process or a continuum
2. Is subjectively defined by the person themselves
3. Is ‘rated’ by the person experiencing the mental health difficulties, who is considered the expert on their recovery.
4. Recovery means different things to different people, although there are aspects that many people share.

Personal recovery has a different focus from clinical recovery, for example in emphasising the centrality of hope, identity, meaning, and personal responsibility [9]. The most widely-cited definition, which underpins most recovery policy internationally, is by Bill Anthony:

*Recovery is a deeply personal, unique process of changing one’s attitudes, values, feelings, goals, skills, and/or roles. It is a way of living a satisfying, hopeful, and contributing life even within the limitations caused by illness. Recovery involves the development of new meaning and purpose in one’s life as one grows beyond the catastrophic effects of mental illness* [10] (p.527)

Since recovery has a personal meaning for each individual, it can be difficult to find a shared definition. In a Delphi study with 381 participants, all of whom had personal experience of psychosis, the highest number of participants agreed on the statements ‘recovery is the achievement of a personally acceptable quality of life’ and ‘recovery is feeling better about yourself’ [11].

Another more succinct definition is ‘Recovery involves living as well as possible’ [12]. This has the merit of focussing attention on what we have in common rather than how we are different: everyone, including both staff and service users, is trying to live as well as possible. It also reflects the reality that we all have challenges which limit our lives, whether related to health problems, social problems (e.g. poverty), interpersonal problems, spirituality, sexuality and so forth. A focus on supporting people to live a life beyond mental health problems has emerged internationally as a key component of the recovery approach.

This distinction between different understandings of recovery has been characterised in different ways: recovery ‘from’ versus recovery ‘in’ [13]; scientific versus consumer models of recovery [14]; clinical recovery versus personal recovery [15] or versus social recovery [16].

It is this second understanding of recovery – personal recovery – which is meant when policies or services refer to supporting recovery, using a ‘recovery approach’ or being based on a ‘recovery model’. It is the meaning of recovery which is embedded in national mental health policy and emerging in practice internationally, including Australia [17], Canada [18], England and Wales [19], Germany [20], Hong Kong [21], Israel [22], Italy [23], the Netherlands [24], New Zealand [25], Northern Ireland [26] and the United States of America [27].

The ultimate arbiter of recovery is therefore the person living with the experience. This is inconvenient from a policy perspective, which has traditionally relied on the judgment of experts to make resource allocation decisions. However, as stated by Robert McNamara, “*the challenge is to make the important measurable, not the measurable important*”. A new evidence base is needed, including new approaches to (for example) establishing benefits entitlement, which locates recovery as a subjective experience rather than as an observable state.

### Message 2: Many people with mental health problems recover

The 2014 review does not give a definitive statement about recovery rates for the three disorders considered. However, the presentation of prevalence data for two of the three disorders opens by summarising findings from relevant recent systematic reviews: a 2013 systematic review by Jääskeläinen and colleagues of outcome in schizophrenia [28] and a 2014 systematic review by Steinert and colleagues of outcome in depression [29].

Our particular interest is in psychosis experiences, so we now discuss the Jääskeläinen schizophrenia review. We anticipate that equivalent concerns also relate to measuring recovery rates in other diagnostic groups.

The Jääskeläinen review summarised the findings of 50 studies of outcome in schizophrenia. It is a well-conducted systematic review, following best practice in reporting [30] and published following peer-review in a reputable academic journal. The take-home message, to quote the conclusion in the abstract (since most readers do not read beyond the abstract), was “*Based on the best available data, approximately, 1 in 7 individuals with schizophrenia met our criteria for recovery*” (p.1296). In other words, the substantial majority of people given a diagnosis of schizophrenia do not recover.

This conclusion is flawed for three reasons: sampling strategy, follow-up period and outcome assessment.

### Problem 1: sampling strategy

The Jääskeläinen review is based on 50 studies. What settings were participants recruited from? The authors laudably include this information in Online supplement Table 1 of the paper, but do not calculate or comment on this aspect in the main paper. (Online supplements give further data not included in a main paper. We suspect that most people accessing a paper read no further than the abstract, and only a tiny portion read not only the full paper but also the online data supplement.) We therefore manually calculated that 23 of the included 50 studies recruited people on admission to hospital, and 7 on discharge from hospital. A further 4 studies recruited from out-patient settings and 12 recruited from a combination of in-patient and out-patient settings. This leaves a total of 4 (8 %) of studies which recruited from the general population. The total population in these general population studies of 434 is 4.8 % of the 8,994 total sample size. In other words, nearly all the included studies identified potential participants who were already in contact with mental health services.

As we discuss later (see Message 6), many people live with psychosis-like experiences outside of mental health services. Their ability to self-manage without attracting the attention of services indicates a lower level of severity and a higher rate of recovery. This means that people with less severe difficulties are systematically less likely to be present in the samples included in the Jääskeläinen review. In other words, the evidence base synthesised in this systematic review indicates a degree of exposure bias and exaggerates the typical level and length of disability associated with the diagnosis of schizophrenia.

### Problem 2: follow-up period

The follow-up length of studies included in the Jääskeläinen review varied. Again, this is not commented on in the main paper, but details are given in Online supplement Table 1. We therefore calculated that 11 studies had follow-up periods of 5 or fewer years, 10 more than 5 but less than 10 years, 10 more than 10 but less than 15 years, 10 more than 15 but less than 20 years, and 9 of 20 or more years. In other words, studies varied enormously in their follow-up periods.

What account was taken of this pattern in the analysis? Almost none: “*In order to describe recovery in studies with different durations of follow-up, we derived the annual recovery rate by dividing the proportion of those who met the recovery criteria by the number of years of follow-up*” (p. 1299). So recovery is assumed to be linear, progressing at a fixed rate per year. No justification is given for this (un-stated) assumption, which is undermined by the review finding that duration of follow-up did not predict recovery estimate. The conclusion this approach leads to is: “*The median annual recovery rate was 1.4 % per annum (Inter-Quartile Range: 0.7 %–2.6 %). With this annual recovery percentage, over 10 years approximately 14 % would be expected to recover*” (p. 1301).

Recovery is not linear. The available empirical evidence indicates that recovery is heavily influenced by context, both social (e.g. social [31] and professional [32] relationships), and psychological (e.g. locus of control [33], wellbeing [34]). Although there is evidence that distinct stages of recovery can be differentiated [9], these stages are not linear [35]. Overall, pooling studies of very different duration into one aggregated analysis is not justified.

### Problem 3: outcome evaluation

How was recovery defined in the Jääskeläinen review? The authors “*attempted to assess recovery as objectively as possible*” (p.1298). Their approach required (1) clinical remission, (2) broader social functioning outcome and (3) at most ‘mild’ symptoms, with persistence of good outcome for a minimum of 2 years. Different measures were used across the studies (summarised in Online supplement Table 1), and included psychopathology and receipt of treatment for clinical remission, and employment, independent living and Global Assessment of Functioning score for social functioning. The authors acknowledge this definition is “*more stringent than the most widely used consensus measure of remission*” (p. 1298), presumably so as to ensure that participants really were recovered and not just in remission.

What recovery rates were found? They ranged across the 50 included studies from 0 % to 58 %. Even to the casual observer, this might raise some concerns about simply pooling the data to produce a single overall estimate of recovery rate. This problem of combining apples and oranges is known in the systematic review trade as heterogeneity, defined as the extent to which there are genuine differences underlying the results of included studies. Dealing with heterogeneity is a standard challenge in systematic reviews. Are the included studies sufficiently similar to be pooled (or ‘meta-analysed’) to produce an overall estimate of recovery rate?

Two approaches are used in review methodology to test for heterogeneity. Visual inspection involves ‘eyeballing’ the data, and the huge variation in recovery rates in Figure 2 of the paper would not give most analysts much confidence that pooling is justified. The second approach is statistical, using a test called the I^2^ statistic, which assesses the percentage of total variation across studies due to heterogeneity. This number ranges from 0 % to 100 %, and the standard rule-of-thumb for this statistic is that 0 % indicates no heterogeneity (i.e. pooling the data is fully justified), 25 % indicates low heterogeneity, 50 % indicates moderate heterogeneity, and 75 % indicates high heterogeneity (i.e. pooling the data is not justified) [36]. In the Jääskeläinen review, the I^2^ score was 99.8 %. Despite this, all the studies were still pooled, to produce the take-home message that 14 % of people with a diagnosis of schizophrenia recover. (We should note that the authors are aware of the issue. They use a particular analysis approach – random effects modelling – to address this issue. However, random effects modelling involves several untested assumptions (e.g. that the recovery rates differ between studies but all follow a distribution, the distribution is normally assumed to be random, etc.), and more generally random effects models do not ‘take account of’ (i.e. deal with) heterogeneity [37]. The authors do attempt to explore sources of heterogeneity in other analyses, but nothing is found – indicating that high uncertainty remains.)

Our overall point is not statistical. Rather, the picture we have painted is that each key decision made in this review leads to a more pessimistic finding. From the entire population of people meeting criteria for a diagnosis of schizophrenia, the focus is on those with more severe problems who are in contact with mental health services. Despite the rather obvious observation that recovery takes time, and often a long time at that, studies of markedly different follow-up periods were treated as equal. The threshold for being ‘recovered’ was deliberately high. Despite being scientifically unjustified, studies were pooled to produce a misleading global recovery proportion.

The conclusion in the Jääskeläinen paper that “*We found no evidence to suggest that we are “getting better” at getting our patients better*” (p.1305) perhaps indicates that the review was conducted from the assumptions of a clinical recovery perspective. The desire to produce a number – an empirically justified answer to the reasonable question ‘How many people recover?’ – may be understandable from this perspective. But it is also toxic. The implicit assumption that ‘mental illness is an illness like any other’ is consistent with a clinical recovery perspective, but has negative consequences on community attitudes [38]; indeed, the evidence that it is a counter-productive message is so strong that it is no longer used in population-level campaigns to reduce mental health-related stigma [39].

From the perspective of personal recovery (the newer and now dominant international understanding of recovery), there is a large knowledge gap. There is only a small and inconclusive empirical evidence base about the relationship between clinical recovery and personal recovery [33, 34, 40, 41]. There has been no long-term epidemiological research (i.e. over decades) to understand how the development of an identity as a person in recovery unfolds over time. A 10-year follow-up study published since the Jääskeläinen review investigated mortality, clinical and social outcomes in 557 individuals with first-episode psychosis, and emphasised the disparity between symptom-based clinical recovery and social recovery [42]. In this analysis, 213 (65 % of 326, missing data 61) were not experiencing psychotic symptoms at follow-up and 140 (46 % of 303, missing data 84) had been symptom free for two years or more, leading the authors to observe that “*the research relating to outcomes in schizophrenia and other psychoses, conducted before the more recent long-term course and outcome studies, has painted an overly pessimistic picture of the clinical course*” (p.384). However, the low rates of employment (22 %) and being in a relationship (32 %) indicated that social exclusion can remain an issue even when clinical recovery has occurred.

A number of long-term (20 or more year) follow-up studies show more than half of people given a diagnosis of schizophrenia experience clinical recovery [43]. At the individual level, more and more people are telling their idiosyncratic stories of recovery, in books [44, 45], web-sites (e.g. https://www.youtube.com/playlist?list=PLE60D451CF87F4324, http://www.scottishrecovery.net/Stories-from-the-narrative-research-project) and in person. Recovery is emerging as much more common than previously understood [46].

Overall, it is not scientifically justified to make a quantitative statement about recovery rates. Those that have been made are definitely under-estimates, and quite possibly major under-estimates, of the true likelihood of recovery.

### Message 3: If a person no longer meets criteria for a mental illness, they are not ill

An embedded assumption in the 2014 review, as in much of mental health practice, is that having once been diagnosed, no longer being diagnosable indicates the person is ‘in remission’ rather than not ill. Whilst it may be true that a person who has had a particular diagnosis (e.g. depression, schizophrenia) has a higher likelihood than the general public of being diagnosable again, the re-framing of ‘well’ in a dichotomous categorisation system as ‘in remission’ is a reasoning bias. ‘Well’ means well!

This reasoning bias reflects assumptions of chronicity and deterioration. For example, in discussing studies of people who experience a single episode with no recurrence, the 2014 review cautions “*…However, the latter percentage comes from a three-year study, which may be too short to accurately detect recurrent episodes*” (p.12). In other words, studies are criticised for being too short to detect relapse, but the possibility of being too short to detect recovery is not considered.

The concept of remission is of course a common health term. It can be helpful, for example in health contexts where long-term surveillance of patients with recurring illnesses is a reasonable use of resources. However, the use of this approach in a mental health context is problematic. One form of stigma is called diagnostic over-shadowing, a process by which physical symptoms are misattributed to mental illness [47]. This is one factor underpinning the scandalous 20-year mortality gap for men and 15-year gap for women between people living with and without mental illness in high income countries [48]. The view of ‘once ill, always ill’ has toxic consequences in a mental health context, and should be challenged.

### Message 4: Diagnosis is not a robust foundation

While the use of diagnostic terms such as ‘schizophrenia’ is valid from a clinical recovery perspective, it must also be emphasised just how contested diagnostic labels are in mental health.

The latest taxonomy is the Diagnostic and Statistical Manual of Mental Disorders Version 5 (DSM-5) [49]. Criteria for schizophrenia are shown in Table 1.

**Table 1:** DSM-5 criteria for schizophrenia

Criteria A to F must all be met
A. Two or more of the following, each present for a significant portion of time during a 1-month period (or less if successfully treated). At least one of these must be (1), (2), or (3): 1. Delusions 2. Hallucinations 3. Disorganised speech 4. Grossly disorganised or catatonic behaviour 5. Negative symptoms B. For a significant portion of the time since the onset of the disturbance, level of functioning in one or more major areas, such as work, interpersonal relations, or self-care, is markedly below the level achieved prior to the onset (or when the onset is in childhood or adolescence, there is failure to achieve expected level of interpersonal, academic or occupational functioning). C. Continuous signs of the disturbance persist for at least 6 months. This 6-month period must include at least 1 month of symptoms (or less if successfully treated) that meets Criterion A (i.e. active-phase symptoms) and may include periods of prodromal or residual symptoms. During these prodromal or residual periods, the signs of the disturbance may be manifested by only negative symptoms or by two or more symptoms listed in Criterion A present in an attenuated form (e.g. odd beliefs, unusual perceptual experiences). D. Schizoaffective disorder and depressive or bipolar disorder with psychotic features have been ruled out because either a) no depressive or manic episodes have occurred concurrently with the active-phase symptoms, or 2) if mood episodes have occurred during active-phase symptoms, they have been present for a minority of the total duration of the active and residual periods of the illness. E. The disturbance is not attributable to the physiological effects of a substance (e.g. a drug of abuse, a medication) or another medical condition. F. If there is a history of autism spectrum disorder or a communication disorder of childhood onset, the additional diagnosis of schizophrenia is made only if prominent delusions or hallucinations, in addition to the other required symptoms of schizophrenia, are also present for at least a month (or less if successfully treated).

Despite being emphasised as central diagnostic features in all previous editions of the DSM, it should be noted that two ‘first-rank symptoms’ (bizarre delusions and voices commenting and/or conversing) have now been removed from this list due to their low reliability. In this respect, diagnostic criteria for schizophrenia to be included in DSM-5 were greatly contested in the years running up to its publication. These issues were not resolved in the scientific community. In the week before DSM-5 was launched, Thomas Insel who heads the US National Institute for Mental Health (the primary funder of mental health research in North America) announced that the NIMH was going to abandon DSM because it dealt only with symptoms and not the genetic and neurological research which he believed ought to be used to define disease entities.

A recent report called ‘Understanding Psychosis’ from the British Psychological Society [50] concluded:

*…reliability remains low for most diagnoses, at least in everyday clinical practice where diagnoses are often made without detailed reference to the official manuals. Clinicians tend to have diagnostic ‘preferences’ and people are often given a range of diagnoses during their contact with mental health services. Research confirms that usage varies between different doctors, hospitals and countries. Even experienced clinicians, who have been given extra training in applying the criteria, only agree on a broad diagnostic category about 50 % of the time.* (p.22)

Other credible commentators have gone further, and argued for the abolition of the term ‘schizophrenia’ altogether [51]. Some of the issues are outlined at http://www.schizophreniainquiry.org.

Why is there such lack of clarity about diagnosis? One explanation is that diagnostic categories in mental health encompass an ever-increasing range of human experiences – the so-called ‘colonisation of the human condition’ [15]. Maddux has characterised the process:

*The social construction of psychopathology works something like this. Someone observes a pattern of behaving, thinking, feeling, or desiring that deviates from some social norm or ideal or identifies a human weakness or imperfection that, as expected, is displayed with greater frequency or severity by some people than others. A group with influence and power decides that control, prevention, or “treatment” of this problem is desirable or profitable. The pattern is then given a scientific-sounding name, preferably of Greek or Latin origin. The new scientific name is capitalised. Eventually, the new term may be reduced to an acronym, such as OCD (Obsessive-Compulsive Disorder), ADHD (Attention-Deficit/Hyperactivity Disorder), and BDD (Body Dysmorphic Disorder). The new disorder then takes on an existence of its own and becomes a disease-like entity. As news about “it” spreads, people begin thinking they have “it”; medical and mental health professionals begin diagnosing and treating “it”; and clinicians and clients begin demanding that health insurance policies cover the “treatment” of “it”. Once the “disorder” has been socially constructed and defined, the methods of science can be employed to study it, but the construction itself is a social process, not a scientific one. In fact, the more “it” is studied, the more everyone becomes convinced that “it” really is “something”.* (p.62) [52]

Causes of ‘mental illness’ are contested. Research disciplines across different modalities (e.g. genetic, biological, psychological, social) commonly exhibit this bias – whatever is found to be influenced by the modality of interest is ‘confirmed’, and whatever is not found to be influenced is ‘unexplained’. This is as a result of the scientific method, which tends to find positive evidence initially even where more robust future investigation finds the apparent relationship to be spurious. Witness the repeated discovery of ‘the gene for X’ which proves not to be substantiated. For example, behavioural genetics aims to establish causal relationships between genes and behaviour [53]. The approach involves identifying genetic influences (e.g. through twin studies), and unexplained variance can then be investigated through studies of shared environment (e.g. through family studies) and non-shared environment (e.g. through adoption studies). This elevates the importance of evidence for genetic influences. The same criticism of course applies to the search for psychological or social causes of mental illness. In the words of Abram Maslow, “I suppose it is tempting, if the only tool you have is a hammer, to treat everything as if it is a nail” [54].

A specific contested point in relation to psychosis experiences is the role of trauma. Scientific evidence is making clear that adverse life events (particularly, but not exclusively, childhood abuse) are experienced by a substantial number of people who go on to develop psychosis (and hence be diagnosed with ‘schizophrenia’ or ‘bipolar disorder’ or other psychosis diagnoses). This was summarised in 2014 [50]:

*Much evidence has now accumulated to suggest that like other mental health problems, psychosis can be a reaction to such stressful events and life circumstances, particularly abuse or other forms of trauma* [55, 56]*. For example, voices may relate to previous trauma which has left difficult feelings and memories that need to be explored and resolved. A review in 2008 found that between half and three-quarters of psychiatric inpatients had been either physically or sexually abused as children* [57]*. Experiencing multiple childhood traumas appears to give approximately the same risk of developing psychosis as smoking does for developing lung cancer* [58]*.* (p. 42)

A second reason is that the mental health system is underpinned by assumptions which give primacy to the genetic and biological and more recently to the psychological, to the neglect of social understandings of mental distress. It has proved difficult for the mental health system, including research approaches, to let go of the assumption that mental illness resides in the person. For example, one response to the above finding that schizophrenia is more common in people who experienced childhood abuse has been to search for the genetic variant which influences response to childhood adversity [59]. Although all scientific research has potential value, the continued effort to individualise socially-caused phenomena – sometimes called ‘responsibilization’ [60] – represents a reasoning bias in mental health research. Other social determinants of mental ill-health include poverty, unemployment and reduced social networks [61]. When these social causes become framed as ‘vulnerability’ factors, i.e. something about the individual, then the real issues of justice, exclusion, power and marginalisation are occluded. We believe that this could come to be considered to be as unacceptable as for example a search for vulnerability to racism in people from ethnic minorities. If the problem is social, then social solutions should be the first recourse. To put it in the language of human rights, ‘Fix society, not people’.

An over-emphasis on diagnosis has adverse consequences. It leads to the development of a separate sub-culture in which specialised rather than mainstream solutions are developed for people with mental health problems who have everyday problems [62]. For example, the person with mental health difficulties who wants a relationship is offered social skills training, or who wants a job is offered pre-vocational training, or who wants to rent an apartment is offered training to be a good tenant. Contrast these responses with how such requests would be responded to in non-clinical social situations.

An orientation towards recovery means starting with an assumption that people with mental illness are first and foremost people [63], so a more useful instinctive response to meeting everyday problems is to support access to mainstream solutions. For example, the evidence that Individual Placement and Support – an approach to supporting people to obtain and maintain a mainstream job – has superior outcomes to pre-vocational training (in which a person is trained to be ready for a job) is overwhelming [64]. A Cochrane review synthesized 18 randomised controlled trials of reasonable quality, and showed 18-month employment rates of 34 % for IPS compared with 12 % for pre-vocational training [65]. For instance, a six-country European randomised controlled trial showed that individual placement and support was superior to the local alternative in each site, in terms of helping people find and maintain paid employment [66]. The same evidence base is emerging in relation to housing, that obtaining a tenancy and providing support to retain it is more effective than pre-tenancy training or first meeting eligibility requirements (e.g. demonstrated sobriety) [46].

Over-emphasising diagnosis also increases mental health-related stigma [67]. Presenting an understanding as ‘how it really is’ reinforces the idea of a meaningful gap between two groups (the ‘mad’ and the ‘sane’). The reality is that we are all damaged in some way.

The unquestioning use of mental illness diagnoses as if they are un-contested and capture meaningful and invariant individual-level diseases is difficult to justify, and may have harmful consequences. It is reasonable for societies to seek defensible and transparent approaches to resource allocation (e.g. welfare benefit entitlement), but the use of diagnosis is a problematic foundation.

### Message 5: Treatment is one route among many to recovery

A clinical recovery world-view is based on what might be described as a surgical metaphor. A person is healthy, then becomes ill (typically evidenced by a disturbance which is not self-corrected in balance – ‘homeostasis’ – in a physical system of the body). Clinical intervention (e.g. surgery, pharmacotherapy) restores the balance, homeostasis is restored, and health returns. From this perspective, treatment is instrumental to improve outcome. Such frameworks have been deemed ‘a technological paradigm’ in that (1) mental health difficulties are believed to arise from disordered processes within the individual, (2) are modelled universally and causally, independent of an individual’s particular context, and (3) resulting interventions are applied and evaluated independently of social/interpersonal values, narratives, and relationships [68]. Such frameworks have been strongly criticised on the grounds that they are poorly equipped for engaging with emotional suffering [69, 70]. Furthermore, as discussed below and previously, empirical evidence from within the paradigm does not support the assumptions upon which it is based.

By contrast, a personal recovery perspective does not assume treatment is needed for recovery. The emerging empirical evidence indicates that individuals experiencing psychosis develop an identity as a person in recovery through a range of routes. A systematic review of 97 studies investigating the experience of recovery identified that one characteristic of the recovery journey is that recovery can occur without professional intervention [35]. A study of the experiences of 381 people living with psychosis found that 82 % agreed with the statement that ‘Recovery is knowing that you can help yourself become better’.

These data are not an argument for reduced provision of mental health services. Mental disorders account for 13 % of global illness burden, and major depression alone is expected to be the largest burden contributor by 2030 [71]. Mental disorders are predicted by 2030 to account for nearly a third of the projected US$47 trillion incurred by all non-communicable health conditions [72]. However, the vast majority of countries allocate less than 2 % of their health budgets to mental health [73]. This creates a ‘treatment gap’ between the 2 % allocated and 13 % needed, which should be reduced not widened. A 58-country survey of this treatment gap demonstrated a global consensus that around 10 % of health spend should be allocated. Scaling up of mental health services is needed, especially in low and middle income countries [74].

Rather, these data support the argument for levelling up – focussing more resources on the wider contributors to recovery. More research is needed, but candidate targets are supporting families in their caring role [75], providing decent housing [76], engaging with employers to help educate them in the work-place adjustments needed by people experiencing mental health problems [77], developing opportunities for people with personal experience of mental illness and recovery to be involved in and lead at all levels in the mental health system [78], political activism by mental health professionals [79], the use of peer-support initiatives [80], and applying societal campaigns such as Time to Change [81] to challenge stigma in the mental health system [82] and wider society. The overarching aim is a re-orienting of the mental health system around the goal of ensuring access for people experiencing mental health problems to the normal entitlements of citizenship [83]. In turn, there is also a strong rationale for dispensing more resources towards primary prevention efforts, e.g. addressing factors like domestic violence, peer bullying, and childhood abuse [56].

Over-emphasising the importance of treatment as the sole route to recovery is both empirically un-justified and maintains many wider contextual hindrances to recovery. Key processes involved in recovery are connectedness, hope, a positive identity, meaning and empowerment [35]. These processes can and do occur outside of the mental health system.

### Message 6: Some people choose not to use mental health services

Many people live with psychosis-like experiences without requiring (or wanting) input from mental health services. For example voice hearing (‘auditory hallucinations’), a cardinal symptom of psychosis, may be often reported amongst those in good psychological health and with no history of mental health service contact. Prevalence estimates vary according to the age range examined and the ways in which voice hearing is defined, but is estimated to reach a median of 13.2 % in the adult general population [84]. Considering that lifetime rates of psychosis are estimated to range from 0.2 % (narrowly defined criteria) to 0.7 % (broadly defined) [85], it is clear that many more individuals hear voices than are diagnosed with psychosis. In turn, voice hearing can show numerous phenomenological similarities in people with and without a need for psychiatric care (e.g. loudness, location, personification, underlying neural activity) [86], with hallucinations found to be associated with delusions in the general population in the same way that they are in psychosis [87]. In this respect, a more consistent predictor of distress and clinical need appears to be emotional responses to, and negative beliefs about, the voices one hears rather than objective presence alone [86], which, at least in some cases, may be influenced by exposure to trauma and social adversity [88–92]. Similarly, distressing persecutory ideation and delusional beliefs show a clear spectrum across the general population [93–95], with paranoia associated with similar psychological factors (e.g. depression, anxiety, interpersonal sensitivity, trauma exposure) in both clinical and non-clinical groups [96]. Taken together, there is plausible evidence that psychosis is a dimensional phenomenon that lies on a continuum with normal human experience rather than a categorical ‘present or absent’ event [97–99].

People living with experiences considered typical of psychosis may not be in contact with mental health services for a range of reasons, including:

- they choose not to have contact with the mental health system
- they are either not distressed by their experiences, or actively value them
- they have a good support network
- they choose not to disclose because they fear being stigmatised if they are given a diagnosis of a mental illness
- they have a non-medical or non-psychological framework for their experiences (e.g. supernatural, spiritual, cultural, technological) and do not identify with models used in mental health services

So the explanations provided in the 2014 review for not being in contact with mental health services (either not knowing about services, or experiencing financial or other access barriers) is incomplete. Many people make choices to live with ‘symptoms’ associated with psychosis outside of the mental health system. Research to understand the influences on this choice, and resulting impact on people’s experiences, should be a priority. However, the implication that all people *should* be in contact with mental health services, and therefore that using mental health services should be either a requirement or an indicator of benefits entitlement, is not justified.

### Message 7: The impact of mental health problems is mixed

The picture conveyed in the 2014 review is that the impact of mental illness is solely and inevitably negative. Without denying the pain and distress of many people who live with mental health difficulties, this perspective is both unjustified and unduly pessimistic. On the contrary, survivor testimony indicates that the process of surviving mental health challenges – including psychosis – can ultimately be transformative, enriching and a source of personal and social growth [44, 100–102]. For example:

*Recovery to me is not only coming to terms with what has happened in my life…but having grown as an individual because of my experiences…I can now look back in time and know that everything that happened helped me to become the person I am today*. [103] (p.46)

*Yes I am MAD! I am not in remission I am not on a trajectory I am not a syndrome I am not delusional. I am living my life. I am living the dream*. [104] (p.178)

*I’m now inspired to speak about the possibilities of recovery, to spread a message of hope, to break down barriers/stigmas… I now believe anything is possible*. [105] (p.246–247)

*For most of my life I have studied the phenomena known as madness, my own and others…Forging friendships with my peers, I found community in our shared experience and our passion for helping fellow travellers. We helped ourselves find meaning when we helped others.* [106] *(p.272)*

*So why would I want anything to do with this illness? Because I honestly believe that as a result of it I have felt more things, more deeply; had more experiences, more intensely; loved more, and been more loved; laughed more often for having cried more; appreciated more the springs for all the winters;… and slowly learned the values of caring, loyalty, and seeing things through.* [107] (p.218)

As noted by Repper, recovery involves the realisation that there are aspects of mental health challenges that can provide growth and positive gain [108]. A particular locus for this perspective comes from the International Hearing Voices Movement, a prominent psychiatric survivor organisation that works to reframe conventional disease models of voice hearing [109, 110]. A central tenet of the Hearing Voices Movement is that voice hearing is a subjectively meaningful experience which, with the right support, can be lived with peacefully and profitably. Correspondingly, the Movement emphasises the possibility of empowerment and psychological growth, as well as exploring the interpersonal and socio-political implications of the identity of ‘voice hearer’ [44, 111–113]. Although the Movement emphasises partnership and alliance between ‘experts by profession’ (clinicians, academics) and ‘experts by experience’ (service-users, their friends and family), many of its prominent members are former psychiatric patients who testify to how their distressing experiences have ultimately informed and augmented their wellbeing (e.g. through a heightened capacity for political engagement, creativity, compassion, fortitude, and self-knowledge) [44, 100, 102, 114].

In turn, the concept of the ‘survivor mission’ captures how one may transform and transcend one’s experiences of adversity in a positive way [115]. For example, mental health workers with their own history of emotional distress may often exhibit greater professional engagement than colleagues without such experiences [116], and the value of employing peer-support workers within services is likewise well-recognised [117]. Similarly, adversities that are closely associated with complex mental health difficulties, such as childhood abuse [118] and violent victimisation [119], can in themselves be a means of ‘posttraumatic growth’ in the sense of inducing positive psychological, social, and interpersonal changes. Taken together such findings attest to the fact that while mental health problems may be devastating and life-changing, they can also lead to a heightened sense of perspective and purpose.

### Summary

This article has identified seven scientifically defensible, relevant and helpful messages about recovery. These messages are intended to be applicable to individuals affected by mental health problems, their family and other informal supporters, and mental health workers.

### Message 1: Recovery is best judged by the person living with the experience

The most important judge of recovery is the person directly affected. Therefore the individual’s values and preferences for specific treatments or other forms of support should be central.

### Message 2: Many people with mental health problems recover

Living well with and beyond ‘illness’ experiences is possible for many people. It involves personal effort and support from others. In relation to benefits entitlement, the criterion of ‘permanent disability’ in a mental health context is toxic, and should not be used. If a time criterion is needed then a duration relating to a reasonable review period should be used, such as ‘expected to persist for at least one year’.

### Message 3: If a person no longer meets criteria for a mental illness, they are not ill

The more a person can develop a rich and layered identity as a person in recovery, rather than a thin identity as a ‘patient’ , the more they will develop resilience and the ability to meet the challenges of life.

### Message 4: Diagnosis is not a robust foundation

Diagnosis is helpful to some but not all people. Therefore it should be used if helpful, but having a different understanding of experiences (e.g. as a response to trauma rather than as an illness) is scientifically justified and for some people can be a turning point on their road to recovery. Diagnosis is a convenient criterion in relation to benefits entitlement, but is contested. Some people choose not to accept their diagnosis, and framing their experience in other ways has a positive influence on their recovery. Making people accept a label in order to access entitlements therefore has negative consequences. New approaches to allocating social resources are needed, which reduce rather than enhance stigma. In the short term, one step towards reducing benefits-related stigma would be to allow disagreement with a diagnosis to be recorded on claim forms without impacting on entitlement. In the longer term, less contested approaches than diagnosis are needed.

### Message 5: Treatment is one route among many to recovery

It is reasonable to expect a full range of established pharmacological, psychological and social interventions to be widely available and competently provided in high income countries. However, some people find other ways forward in their life – there is more than one road to recovery.

### Message 6: Some people choose not to use mental health services

People choose not to use mental health services for a range of reasons. Of these, some would benefit from them, and others live well outside of services.

### Message 7: The impact of mental health problems is mixed

Recovery may not mean getting one’s previous life back – none of us can go backwards – but many people identify that the experience of mental ill-health has unexpected benefits.

All discourses, including scientific ones, are vulnerable to confirmation bias, in which evidence supporting the discourse is more likely to be noticed and accepted than evidence disconfirming the discourse [120]. We believe that the dominant discourse within mental health is one of limitations, in which clinical and research skills are more oriented towards identifying for example disabilities than strengths, risk factors than protective factors, vulnerability than resilience, and threats than opportunities. Such a discourse is more likely to produce pessimistic results. As our goal in this selective review was to provide a balancing rather than balanced perspective, we therefore included more quotes and links to narrative web-sites than typical in scientific papers. Our aim was to increase the visibility of evidence from the subjective experience of individuals. Future reviews should use methodologies such as narrative synthesis [121] to integrate the full range of nomothetic evidence from studies of groups and idiographic evidence from studies of individual [122], with the aim of providing a balanced and helpful appraisal of the empirical evidence about recovery.

## Abbreviations

DSM: Diagnostic and Statistical Manual of Mental Disorders
IPS: Individual Placement and Support
